# Increased Serum and Urinary Oxytocin Concentrations after Nasal Administration in Beagle Dogs

**DOI:** 10.3389/fvets.2017.00147

**Published:** 2017-09-05

**Authors:** Andrea Temesi, Julianna Thuróczy, Lajos Balogh, Ádám Miklósi

**Affiliations:** ^1^Department of Ethology, Eötvös Loránd University, Budapest, Hungary; ^2^Animal Health Center Budafok, Budapest, Hungary; ^3^National Public Health Center, National Research Directorate for Radiobiology and Radiohygiene, Budapest, Hungary; ^4^MTA-ELTE Comparative Ethology Research Group, Budapest, Hungary

**Keywords:** dog, intranasal, oxytocin, secretion, cortisol

## Abstract

In recent years more and more studies have revealed the effect of extraneous oxytocin on the social behavior of dogs. The distribution of administered oxytocin in different physiologically relevant compartments is important because this knowledge forms the basis for the timing of behavior tests after the administration. Most behavioral studies rely on the non-invasive intranasal application of oxytocin. The aim of this study was to determine the time course of intranasal administered oxytocin secretion into blood and urine and also establish a connection between intranasal received oxytocin and urinary cortisol in dogs. In our experiment, four dogs received three puffs, 12 IU intranasal oxytocin treatment, two dogs received three puffs intranasal placebo treatment. Blood and urine samples were collected immediately prior to the administration then regularly during 4 h. After nasal oxytocin application, the serum oxytocin concentration increased, reached a maximum 15 min after the treatment and then rapidly returned to baseline levels 45 min later. The peak urinary oxytocin concentration occurred between 45 and 60 min after administration and returned to baseline levels slowly. We found considerable differences among individuals in the secretion of oxytocin in both the serum and the urinary oxytocin concentration measurements. Our results confirm that intranasally administered oxytocin passes into the blood stream. The time course of intranasally administered oxytocin secretion is similar to the time course of intravenously administered oxytocin secretion, and the peak values are also similar in both the serum and the urinary oxytocin concentration measurements, although there are large individual differences.

## Introduction

Beside the reproductive functions, oxytocin plays an important role in the regulation of social behavior (pair bonding, sexual and maternal behavior, peer recognition, and social memory) [for reviews, see Ref. (1, 2)]. Recent studies have revealed the relevance of oxytocin in human bonding, trust and in some aspects of human social cognition including social perception, emotion recognition, sensitivity to others’ experiences, and prosocial behaviors [for reviews, see Ref. (3, 4)].

The manipulation of the oxytocin system is a possible tool for changing sociocognitive performance. Human studies reported some beneficial effects of oxytocin on social attention and emotion recognition in autistic individuals, and reduction of social anxiety in patients suffering from social phobia and borderline personality disorder (5–9).

Similar therapeutic value can be relevant for companion animals with behavioral problems. There are millions of dogs and cats relinquished to shelters after abandonment or abuse. Medical treatment as a complementary therapy to behavioral intervention may help the social integration of these companion animals to rejoin human families.

In recent years, increasing attention has been paid to the effect of extraneous oxytocin on the social behavior of dogs (10–14).

There have been some doubts whether and how intranasally administered oxytocin reaches specific brain areas. Thus, several studies attempted to measure oxytocin concentration and the time course of this effect in the brain, plasma, urine, and saliva. In male rats and mice, increased oxytocin content was measured in microdialyzates from both the left amygdala and the right dorsal hippocampus after nasal application of oxytocin (15). This study showed that nasally administered oxytocin reaches behaviorally relevant brain areas and these changes are paralleled by changes in plasma oxytocin concentrations. The pharmacokinetics of intranasally administered oxytocin was also investigated in human saliva, blood, and cerebrospinal fluid (CSF) (16–19), in dog plasma and urine (10), in rhesus macaque blood and CSF (20–22), and in pig CSF (23). Knowing the distribution of oxytocin in different physiologically relevant compartments is important because this knowledge forms the basis for the timing of behavior tests after the administration of oxytocin. So far in behavior studies intranasal administration is followed by a 40 min waiting period that is presumed to be necessary for central oxytocin levels to reach a plateau based on the vasopressin measurements in the CSF (24).

The short-term use of intranasal oxytocin administered to humans in dosage up to 40 IU results in no adverse reactions (25). Only few studies have investigated the effects of long-term administration of oxytocin in humans (26–28). Some researchers have also revealed dosage dependent effects of intranasally administered oxytocin (29, 30) that should be considered in the case of regular use. Some negative effects of oxytocin were also documented (26, 31). For example, in male prairie voles (*Microtus ochrogaster*), long-term developmental treatment with low doses of intranasal oxytocin resulted in a deficit in partner preference behavior (32).

Intracerebral oxytocin inhibits the stress-induced activity of the hypothalamic–pituitary–adrenal axis responsiveness, thus oxytocin may have an inhibitory influence on stress-responsive neurohormonal systems under physiological condition (33). Some studies found that suckling stimulation produced a significant increase in plasma oxytocin levels and a significant decrease in plasma cortisol concentrations (34–37). Human research demonstrated that oxytocin infusion decreases plasma cortisol (38, 39). In contrast, no significant alterations in cortisol were observed following intranasal oxytocin administration (16). After positive human–dog interaction both species showed significant increases in plasma oxytocin, but only human participants showed a significant decline of cortisol. No similar change occurred in dogs (40). Little increase was found in urinary cortisol levels after intravenous oxytocin injection in dogs, and exercising increased urinary oxytocin concentrations, but had no effect on urinary cortisol (41). In this study, the authors argued that increased oxytocin may have inhibited a cortisol response despite other observations that exercise increases cortisol concentration [e.g., Ref. (42, 43)]. These discrepancies suggest that the inhibitory effect of oxytocin on cortisol secretion may not always be observed.

Most behavioral studies rely, however, on the non-invasive intranasal application of oxytocin. Thus, the aim of this study is to determine the time course of oxytocin secretion into blood and urine after intranasal administration and also establish a connection between blood oxytocin and urinary cortisol in dogs. We also measured the cortisol/creatinine ratio (C/C ratio) to control the water metabolism of the dogs. Our study is a follow up experiment to the work of Mitsui et al. (41) who used i.v. administration. In general, we have expected similar pharmacokinetic effect of the drug thus we included only a restricted sample of dogs based on ethical considerations.

## Materials and Methods

### Subjects

Six (three males, three females, mean age: 2.75 ± SD = 1.13; SEM = 0.46) healthy intact beagle dogs bred by the National Public Health Center, National Research Directorate for Radiobiology and Radiohygiene (OKK-OSSKI) were involved in the study. The animals were not given any medication prior to the study, and they have not previously participated in similar research. Identification of individuals has occurred on the basis of chips’ last four digits. Four dogs represented randomly the experimental group (724, 233—males, 825, 827—females), two beagles were assigned to the control group (760—males, 9,695—females).

### Ethical Statement

Research was done in accordance with the Hungarian regulations on animal experimentation and the Guidelines for the use of animals in research described by the Association for the Study Animal Behavior. Ethical approval was obtained from the National Animal Experimentation Ethics Committee [Ref. No.: TTK/12187/1 (2016), Cert. No.: ELTE-AWC-016/2016].

### Experimental Procedure

#### Preparation

Hair was sheared above the *v. cephalica antebrachii*, and the skin was cleaned by alcoholic disinfectant solution. Venous catheter were placed and fixated in all dogs. Urinary bladder was emptied by urethral catheter just before initiation of testing. Urine samples of bitches were collected by Foley catheter, and samples of males were collected by Buster male catheter. Test was started within 30 min after the placement. All dogs were familiar with the experimental room.

#### Treatment

Four dogs (two males and two females) received three puffs, 12 IU (4 IU/puff) intranasal oxytocin (Syntocinon, Novartis) treatment. This amount is regularly applied in dogs [e.g., Ref. (11, 12)] and it is half of the amount typically used in human studies [e.g., Ref. (44, 45)]. Two dogs (one male and one female) received three puffs intranasal placebo (0.9% NaCl solution) treatment. No force was applied during treatments. The assistant who was familiar to the dogs, held the head of the animals gently for the time of the application. We did not miss any administration. Dogs were kept in individual cages in a silent room after the administration, between the sample collections water was available *ad libitum* and animals saw each other during the examination period.

#### Collection of Samples

Blood and urine samples were collected immediately prior to the administration (Time 0), then every 15 min between 0 and 2 h (Time 15, 30, 45, 60, 75, 90, 105, and 120) and followed by 30 min sampling up to 4 h following the administration (Time 150, 180, 210, and 240). Sample collection did not take longer than 3–4 min per dog per occasion and was done by a trained veterinarian and assistant who were familiar to the subjects on daily basis. Blood samples (0.5–0.7 ml) were collected in tubes kept on ice without anticoagulant containing aprotinin and just after coagulation were centrifuged at 1,000 × *g* for 15 min at 4°C. Coagulation was done for about 20 min. The ice-keeping period was depending on coagulation and occupation of centrifuge but not more than 40 min. Ice was supplied as needed. Serum samples were frozen at −80°C and urine samples were kept at −20°C until the measurement.

#### Immunochemistry

Serum and urinary oxytocin concentrations were measured by competitive ELISA kits. Samples were extracted as it is prescribed in assay procedure (Oxytocin ELISA DE-3117, Demeditec Diagnostics GmbH, Germany; detection range: 15.6–1,000 pg/ml; reactivity: human, all animals). An equal volume of 0.1% trifluoroacetic acid in water (TFA–H_2_O) was added to the sample and centrifuged at 10,000 × *g* for 15 min at 4°C. The supernatant was filtrated by Sep-Pak column (Waters, Hungary) pretreated with acetonitrile and TFA–H_2_O. The sample was eluted from the column with use of acetonitrile and TFA–H_2_O mixture. The sample was evaporated by centrifugal vacuum concentrator (Labconco, USA). The samples were reconstituted with assay buffer occurred just before the measurement. The extraction efficiency was determined by 200 pg/ml oxytocin spiked, extracted, paired samples. The assay procedure describes the way of sample dilution with Standard 0, and thus we accepted the linearity as it is described in the product information leaflet. Assay precision is described with 10.2% intra-assay and 11.8% inter-assay CV, sensitivity 15.0 pg/ml.

Urinary creatinine concentrations were measured by colorimetry (Creatinine, Normachem, Hungary), 2.2% intra-assay and 3.89% inter-assay CV, sensitivity 2.3 mmol/l. In the initial assays, some specimens (six) were found to contain more oxytocin and creatinine than the highest standard. Therefore, these specimens were diluted with Standard 0 in 1:4 ratio, and final concentration was calculated by four times multiplication of measured result.

Urinary cortisol concentrations were measured by Cortisol ELISA (DRG International Inc., USA), 3.2% intra-assay and 7.7% inter-assay CV, sensitivity 6.9 nmol/l.

### Data Analysis

**Figure 1:**
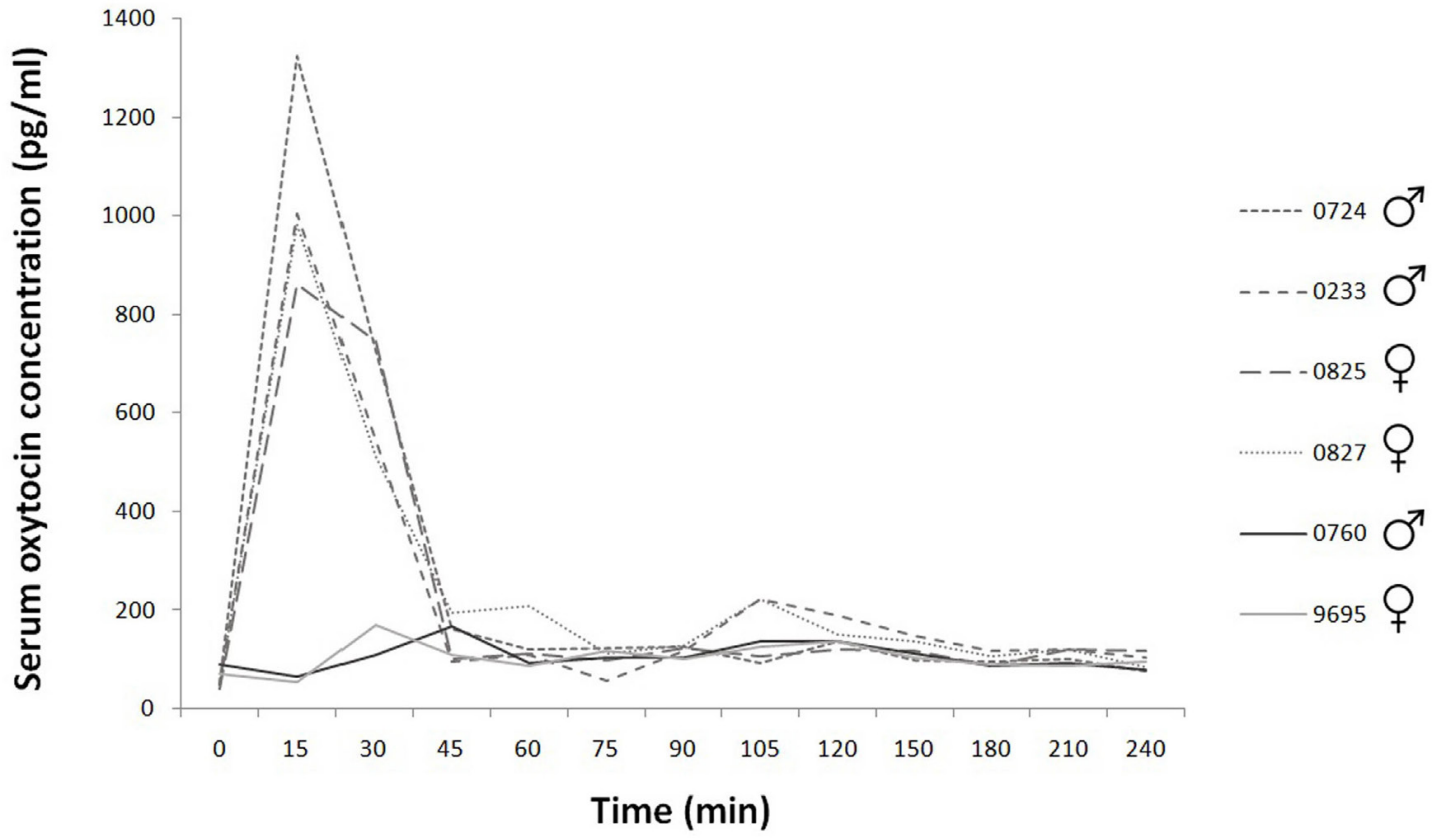
Time course of individual serum oxytocin concentrations during the 240 min. Experimental group: dashed line; control group: continuous line.

In the case of urinary oxytocin and cortisol concentration measurement, there are missing data at some time points. There was no urine in the urinary bladder in these cases. Due to missing data, the low number of subjects, and the individual differences, the possibility of statistical analysis of these data is limited. In addition to the descriptive analysis, statistical analyses were carried out using SPSS (version 22.0.0). Based on visual inspection of serum oxytocin concentration changes (see Figure 1), we focused on the data between 15 and 60 min and used linear mixed models including time and treatment and their two-way interaction as fixed factors, and dog ID as a random term.

## Results

### Serum Oxytocin Concentration

The basal concentration was 58.60 ± SD = 18.27; SEM = 7.46 pg/ml (*N* = 6). After nasal oxytocin application, the serum oxytocin concentration increased, reached a maximum 15 min after the treatment, and then rapidly returned to baseline levels (Time 45) (Figure 1). We found treatment × time interaction to have significant effect both on absolute and relative (i.e., compared to baseline) concentrations [absolute: treatment × time *F*(4,16) = 30.35, *p* < 0.001; driven by differences between relative oxytocin levels at *T* = 30 vs *T* = 60: *b* ± SE = 444.60 ± 112.70 pg/ml, *t*_16_ = 3.95, *p* = 0.001 and *T* = 15 vs *T* = 60: *b* ± SE = 936.28 ± 112.70 pg/ml, *t*_16_ = 8.31, *p* < 0.001 in oxytocin treated as opposed to control group]. Similar results were obtained when concentrations at the start of the experiment (time = 0) were subtracted from concentrations measured at later times (15, 30, 45, and 60 min) following oxytocin or placebo treatment [relative: treatment × time *F*(3,12) = 26.78, *p* < 0.001; driven by differences between relative oxytocin levels at *T* = 30 vs *T* = 60: *b* ± SE = 444.60 ± 125.56 pg/ml, *t*_12_ = 3.54, *p* = 0.004 and *T* = 15 vs *T* = 60: *b* ± SE = 936.28 ± 125.56 pg/ml, *t*_12_ = 7.46, *p* < 0.001 in oxytocin treated as opposed to control group].

### Urinary Oxytocin Concentration

**Figure 2:**
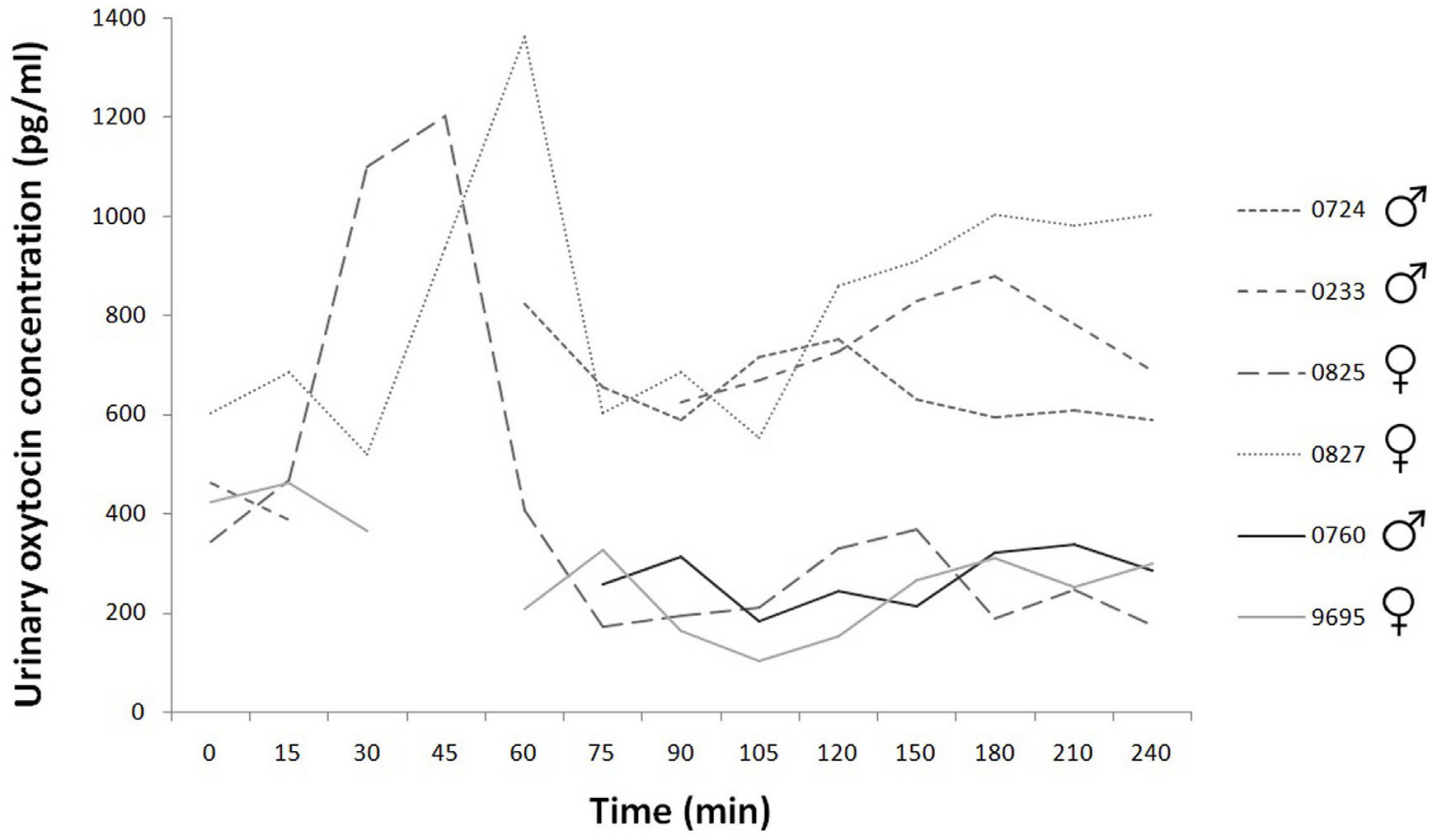
Time course of individual urinary oxytocin concentrations during the 240 min. Experimental group: dashed line; control group: continuous line.

Based on the existing data, the basal concentration was 429.29 ± SD = 113.76; SEM = 50.87 pg/ml (*N* = 5). The peak urinary oxytocin concentration after nasal oxytocin application in female dogs occurred between Time 45 and Time 60 and returned to baseline levels slowly. There are missing data at some data point mainly for male dogs (urinary oxytocin: 78 sample collection, 12 missing data), because the urinary bladder was empty. Large individual differences are perceivable in the decay of the peak values for the two females. The values of the placebo treated dogs’ urinary oxytocin concentrations are clearly distinguishable from the values of the oxytocin treated dogs, there are not similar high peaks for the former (the concentrations remain low, under 500 pg/ml) (Figure 2).

### Urinary Cortisol Concentration

**Figure 3:**
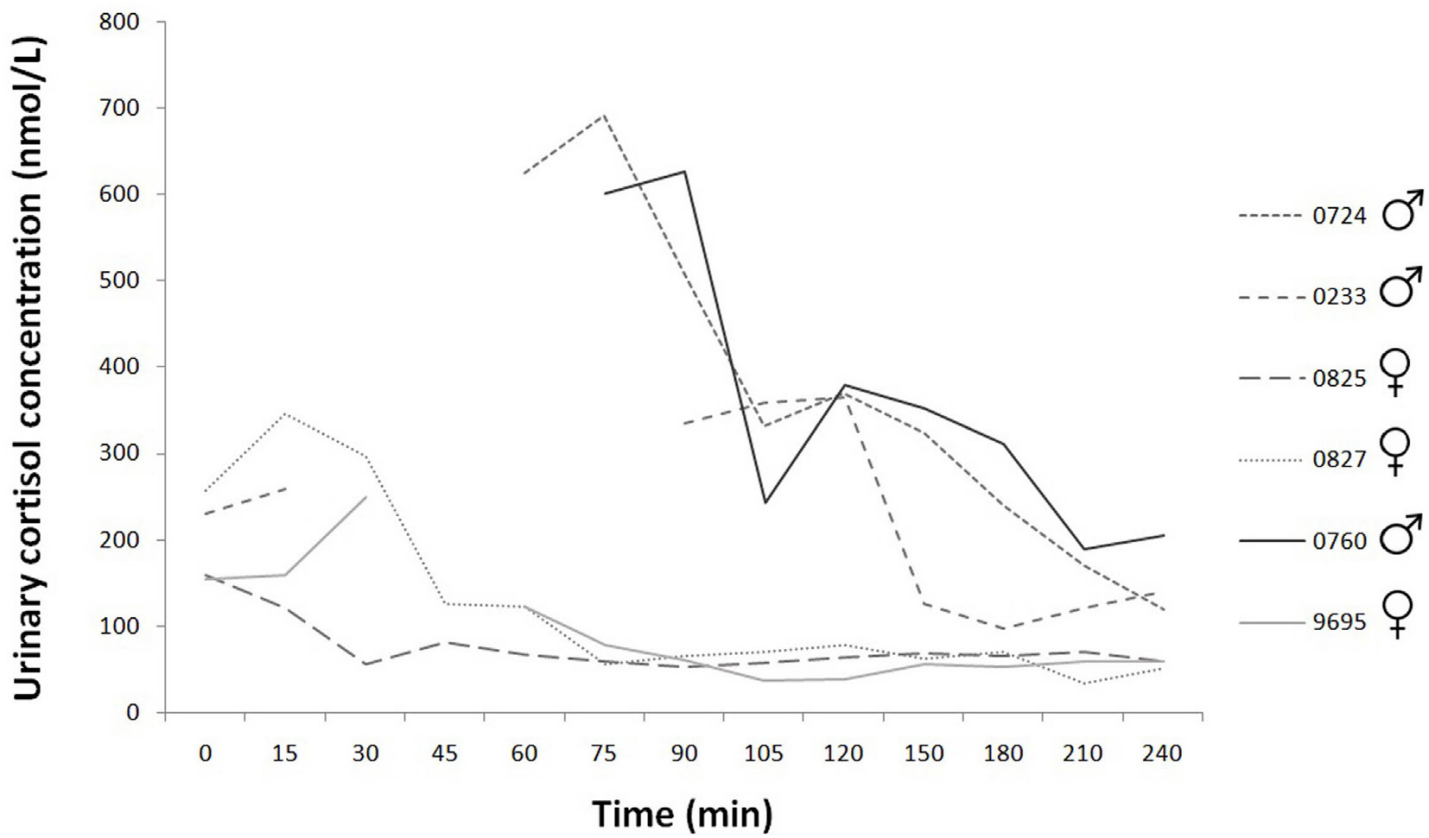
Time course of individual urinary cortisol concentrations during the 240 min. Experimental group: dashed line; control group: continuous line.

Based on our data, the basal concentration was 189.56 ± SD = 50.90; SEM = 22.76 pg/ml (*N* = 5). Mainly in the case of male dogs there are missing data points (urinary cortisol: 78 sample collection, 12 missing data), there was no urine in their bladder at these times. However, according to the available data, the concentration values are clearly higher in male dogs than female dogs (Figure 3).

### Cortisol/Creatinine Ratio (C/C Ratio)

**Figure 4:**
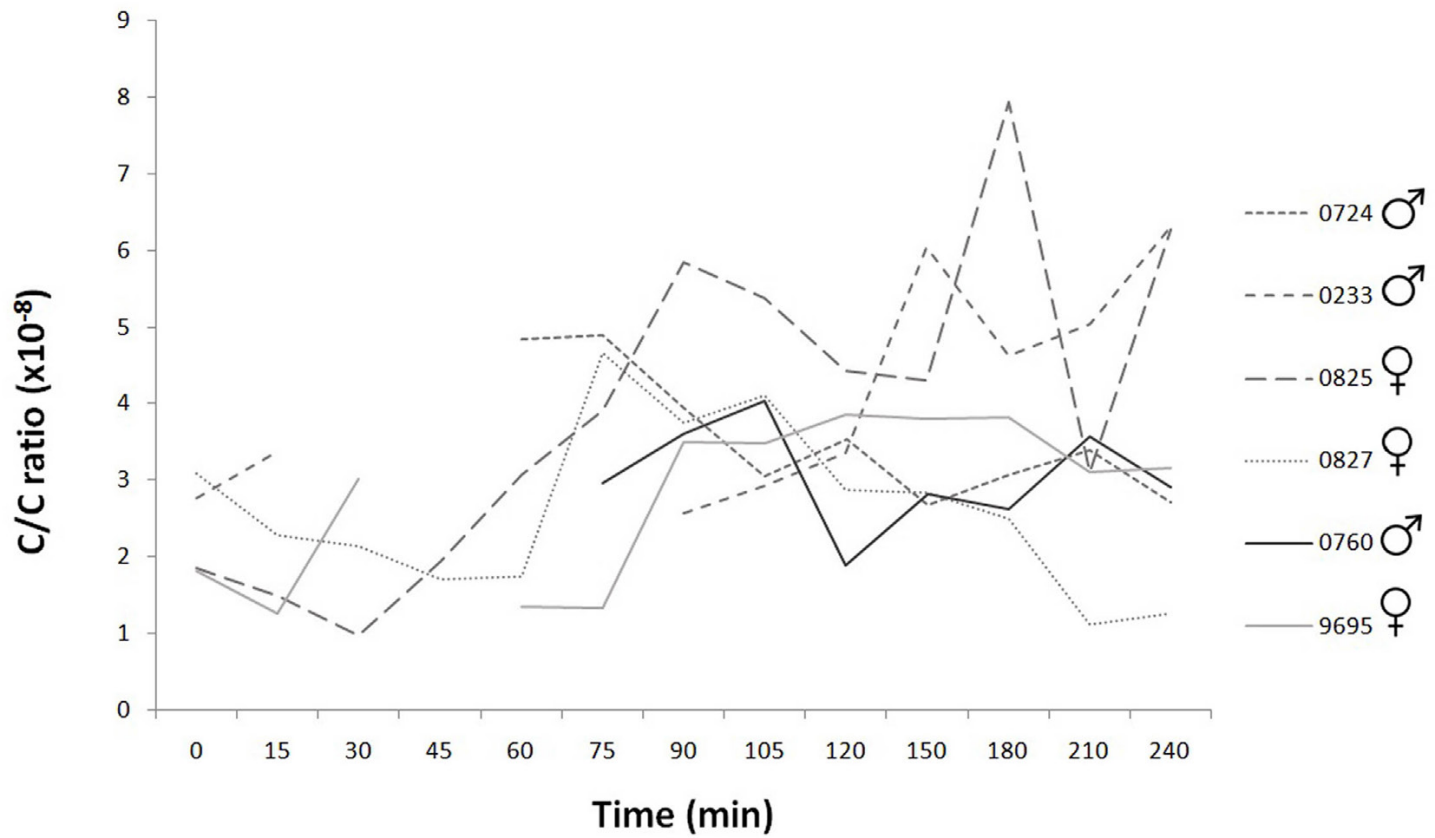
Time course of individual cortisol/creatinine ratios during the 240 min. The C/C ratio of healthy dog is under 10 × 10^−6^. The values did not exceed this limit during the examination period in any subjects.

The C/C ratio indicated the normal water metabolism, so the measured oxytocin and cortisol concentrations were not influenced by any of the factors related to the water excretion (Figure 4).

## Discussion

The aim of this study was to measure the time course of intranasal administered oxytocin secretion into blood and urine and investigate the connection between intranasal received oxytocin and urinary cortisol in dogs.

After three puffs, 12 IU intranasal oxytocin, the serum oxytocin concentration increased, reached a maximum 15 min following the treatment then rapidly returned to baseline levels (Time 45). Similar findings were reported in dogs; however, in that study a higher dose of oxytocin was used (10). This explains that the oxytocin concentration was significantly higher in treated animals than in controls even after 90 min. Our results are more likely in line with the time lapse of plasma oxytocin concentrations were measured in dogs after the animals were injected with exogenous oxytocin intravenously (41). Human studies also found sharp increase in plasma oxytocin concentrations peaking at 10–40 min after treatment, and the levels returned to baseline only at 75–150 min after administration (16, 17, 19, 46). Our results confirm that intranasally administered oxytocin passes into the blood stream. The concentration increases rapidly for a limited time but the time window differs among studies and possibly also among individuals (17).

Recent studies compared plasma oxytocin concentrations after using either intranasal spray or a nebulizer (20, 21). Only the nasal spray oxytocin administration resulted in significant increases in peripheral oxytocin. Further, the concentration returned to the baseline level sooner after nebulizer, this time course is similar to our results after treatment with nasal spray.

In the case of urinary oxytocin and cortisol concentration measurement, we could not obtain data at some time points because there the urinary bladder was empty. Each handling induces some excitation in dogs; water consumption is influenced by excitation also. Animals had free access to water but they did not drink enough in an “interesting” situation. Total emptying of urinary bladder was needed for correct measurement of excretion, but the decreased interest for drinking limited the urination. Due to these missing data, the low number of subjects, and the individual differences, the possibility of statistical analysis was limited. Nevertheless, the peak urinary oxytocin concentration occurred between Time 45 and Time 60 and returned to baseline levels slowly. Similar findings were presented after intravenous oxytocin injection (41). According to the descriptive analysis, we found considerable differences between individuals in the secretion of oxytocin in both the serum and the urinary oxytocin concentration measurements.

Many studies reported elevated oxytocin levels in the CSF after intranasal oxytocin treatment (19, 20, 23). This effect has been explained by three non-exclusive mechanisms (47–49): (1) direct passage of exogenous oxytocin through the BBB; (2) indirect feedback signals from the periphery could stimulate endogenous oxytocin secretion; and (3) oxytocin utilizes specific connections between the nasal cavity and the brain provided by the olfactory and trigeminal nerves. The first possibility was questioned by several early studies (50, 51). The contribution of the second mechanism was made less likely by showing that intranasally administered exogenous (D_5_-deuterated) oxytocin increased labeled oxytocin in the CSF but did not change plasma and CSF endogenous oxytocin concentrations (22). Thus, it is most likely that intranasal oxytocin reaches the brain directly by various extracellular mechanism involving perineuronal channels, perivascular spaces, or lymphatic channels (52, 53).

Although we know that intranasally administered oxytocin passes into the CSF, further research is needed to reveal whether the central access is responsible for the neurobehavioral effects demonstrated by previous studies or peripheral pathways also contribute to the observed effects. At the moment, the distribution of administered oxytocin in the brain is also unknown (22).

Treatment did not change urinary cortisol concentration; however, according to the available limited data, the concentration values are higher in male dogs than female dogs. The venous cannula and the urethral catheter placement, fixation and saliva sampling did not cause any significant, increased pain, and stress to the normal veterinary intervention. Nevertheless, one may assume that the sexes reacted differently to the handling procedure. Similar sex difference in cortisol responses to psychological stress was also found in humans (54, 55).

The serum estrogen and progesterone concentrations of bitches vary depending on estrous stage, in contrast to testosterone in males, which is roughly constant (56). In addition, the pathway of steroid hormone metabolism in mammals is influenced and limited by different enzymatic effects. The higher cortisol levels in males can be explained by the multistep cascade mechanism of steroids, cortisol is also newly formed from its breakdown products. Males generally show an increased cortisol regeneration enzyme activity (57). This may also explain the higher cortisol levels in males. However, it is possible that there is difference between the effect of endogenous and the effect of exogenous oxytocin on cortisol secretion.

Due to the invasive nature of such research (and the follow up aspect of this study), we [see also Ref. (10, 17, 23, 41)] limited sample size following animal welfare recommendations (58). However, the sampling success—especially in complex living organisms—cannot be guaranteed. The lack of data and the small number of subjects precluded partly the statistical analysis. Large individual and methodological differences among oxytocin studies warrant further independent investigations.

In addition to the possible individual differences, the pharmacokinetics of oxytocin might also differ in females and males and in different species. More investigations are needed to determine safe and effective doses for chronic intranasal oxytocin both in different sexes and in different species.

## Conclusion

In summary, our results confirm that similarly to i.v. application, intranasally administered oxytocin passes into the blood stream. The time course of intranasally administered oxytocin secretion is similar to the time course of intravenously administered oxytocin secretion, and the peak values are also similar in both the serum and the urinary oxytocin concentration measurements, although there are large individual differences.

## Ethics Statement

Research was done in accordance with the Hungarian regulations on animal experimentation and the guidelines for the use of animals in research described by the Association for the Study Animal Behavior (ASAB). Ethical approval was obtained from the National Animal Experimentation Ethics Committee [Ref. No.: TTK/12187/1 (2016), Cert. No.: ELTE-AWC-016/2016].

## Author Contributions

AT: substantial contributions to the conception and design of the work; the acquisition, analysis, and interpretation of data for the work; drafting the work and revising it critically for important intellectual content; final approval of the version to be published; agreement to be accountable for all aspects of the work in ensuring that questions related to the accuracy or integrity of any part of the work are appropriately investigated and resolved. JT and LB: substantial contributions to the design of the work; the acquisition and analysis of data for the work; revising the work critically for important intellectual content; final approval of the version to be published; agreement to be accountable for all aspects of the work in ensuring that questions related to the accuracy or integrity of any part of the work are appropriately investigated and resolved. ÁM: substantial contributions to the conception of the work; the interpretation of data for the work; revising the work critically for important intellectual content; final approval of the version to be published; agreement to be accountable for all aspects of the work in ensuring that questions related to the accuracy or integrity of any part of the work are appropriately investigated and resolved.

## Conflict of Interest Statement

The authors declare that the research was conducted in the absence of any commercial or financial relationships that could be construed as a potential conflict of interest.
